# Engagement in Assisted Living During the COVID-19 Pandemic: Challenges and Promising Practices.

**DOI:** 10.1093/geroni/igab046.1334

**Published:** 2021-12-17

**Authors:** Victoria Bartoldus, Youngmin Cho, Janelle Perez, Jing Wang, Stephanie Palmertree, Anna Beeber

**Affiliations:** 1 University of North Carolina at Chapel Hill, Chapel Hill, North Carolina, United States; 2 University of North Carolina at Chapel Hill School of Nursing, Chapel Hill, North Carolina, United States; 3 Fudan University, Chapel Hill, North Carolina, United States; 4 University of North Carolina at Chapel Hill, UNC Chapel Hill, North Carolina, United States

## Abstract

The “lockdown” in assisted living (AL) from the COVID-19 pandemic has physically isolated residents from the outside world and affected resident and family engagement in care. This presentation outlines a content analysis of qualitative semi-structured telephone interviews conducted from April 2020 with 105 AL staff, residents, and family members exploring COVID-19 experience/restrictions and engagement during the pandemic. Analysis revealed AL families and residents expressed difficulties with COVID-19 visiting and distancing restrictions, reduced family visitations, discontinuity of care, and worries about COVID-19 infection. Staff/administrators expressed uncertainty about lack of knowledge about COVID-19, worries about transmission, and if staff will get exposed outside of work. Promising factors include enhanced communication between staff and families regarding care, improved virtual communication, creative strategies to socially engage residents, and improved infection control practices and staff training. The presentation discusses the implications of the findings for future research, policy, and practice.

